# Supplementary material for: brca2-mutant zebrafish exhibit context- and tissue-dependent alterations in cell phenotypes and response to injury
Source: Sci Rep. 2022 Jan 18;12:883. doi: 10.1038/s41598-022-04878-9 (PMC8766490; doi:10.1038/s41598-022-04878-9)
Supplement: Supplementary file 1 — Supplementary Information. [file 41598_2022_4878_MOESM1_ESM.pdf]

brca2-mutant zebrafish exhibit context- and tissue-dependent alterations in cell phenotypes and response to injury

Vassili A. Kouprianov[1], Aubrie A. Selmek[2], Jordan L. Ferguson[3], Xiaokui Mo[4], and Heather R. Shive[2]

1. KBI Biopharma, Durham, NC, USA
2. Department of Veterinary Biosciences, College of Veterinary Medicine, The Ohio State University, Columbus, OH, USA
3. Department of Molecular Biomedical Sciences, College of Veterinary Medicine, North Carolina State University, Raleigh, NC, USA
4. Department of Biomedical Informatics, College of Medicine, The Ohio State University, Columbus, OH, USA

Correspondence to: Dr. Heather Shive; shive.5@osu.edu

Supplementary Information

1. Supplementary Figure Legends
2. Supplementary Figures S1-S3
3. Supplementary Tables S1-S4

Supplementary Figure S1. Optic nerve pathway anatomy, delineation of uninjured and injured optic nerves, and RNA probe and antibody validations and negative controls for *in situ* hybridization and immunohistochemistry experiments. (a) Representative histologic section of the optic nerve pathway after unilateral optic nerve injury. **(b,c)** Representative examples of the uninjured (b) and injured (c) optic nerve. The portion of the nerve used for quantification of total cellularity is outlined in black. In panels d-j, aeas boxed in lettered figures are shown to the right. **(d)** *blbp* expression in the brain (red chromogen). **(e)** sox2 expression in the brain (brown chromogen). Red and yellow arrows indicate positive and negative nuclei, respectively. Yellow asterisk, nucleated red blood cells within the choroid rete (negative for sox2 expression). **(f)** sox10 expression in the brain (brown chromogen). Red and yellow arrows indicate positive and negative nuclei, respectively. Yellow asterisk, nucleated red blood cells within the choroid rete (negative for sox10 expression). **(g)** lcp1 expression in the spleen (purple chromogen). **(h)** Negative control probe for RNA *in situ* hybridization experiments (red chromogen). **(i)** Secondary-only antibody control with hematoxylin counterstain for sox2 and sox10 immunohistochemistry experiments (brown chromogen). **(j)** Secondary-only antibody control with methyl green counterstain for lcp1 and krt18 immunohistochemistry experiments (purple chromogen). Ret, retina; SM, skeletal muscle; CR, choroid rete; ON, optic nerve; UI, uninjured; I, injured. Scale bar = 50 µm (panels a); 20 µm (panels b, c).

Supplementary Figure S2. Quantitative analysis of sox2-, sox10-, and krt18-expressing cells in the zebrafish optic nerve after unilateral optic nerve injury. (a) Representative examples of sox2 expression (brown chromogen), a marker for neural stem cells, in the injured and uninjured optic nerve. Red and yellow arrows indicate positive and negative nuclei, respectively. **(b,c)** Quantitative analysis of sox2-positive cells in the optic nerve three days (b) and two weeks (c) post-injury. **(d)** Examples of sox10 expression (brown chromogen), a marker for neural crest progenitor cells, oligodendrocytes, and Schwann cell precursors, in the injured

and uninjured optic nerve. Red and yellow arrows indicate positive and negative nuclei, respectively. **(e,f)** Quantitative analysis of sox10-positive cells in the optic nerve three days (e) and two weeks (f) post-injury. **(g)** Representative examples of krt18 expression (purple chromogen), a marker for reactive astrocytes in zebrafish optic nerve, in the injured and uninjured optic nerve. **(h,i)** Quantitative analysis of krt-18 positive area in the uninjured versus injured optic nerve three days (h) and two weeks (i) post-injury. Ret, retina; CR, choroid rete; ON, optic nerve, UI, uninjured; I, injured. Scale bar = 100 µm (panels a, d); 50 µm (panel g).

Supplementary Figure S3. Semi-quantitative analysis of *blbp* and sox2 expression in zebrafish ocular tumors. Expression of *blbp* (red chromogen) and sox2 (brown chromogen) is low or absent in the majority of ocular tumors regardless of optic nerve injury (ONI) status. Tumor invasion and subsequent optic nerve/retinal disruption induces marked expression of *blbp* in the retina. Arrows indicated dispersed melanin pigment within the tumor derived from the disrupted RPE. Ret, retina; RPE, retinal pigmented epithelium. Scale bar = 50 µm.

Supplementary Figure S1

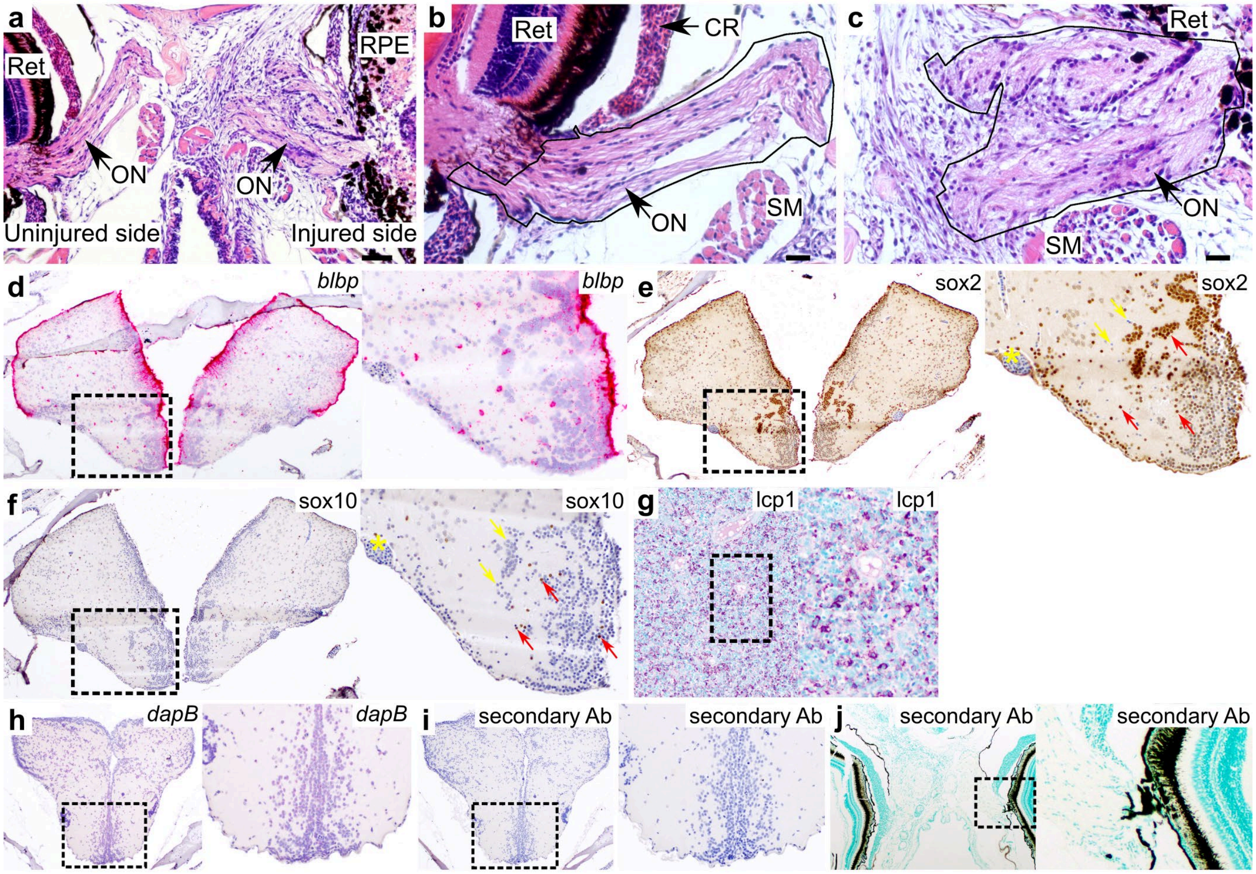

Supplementary Figure S2

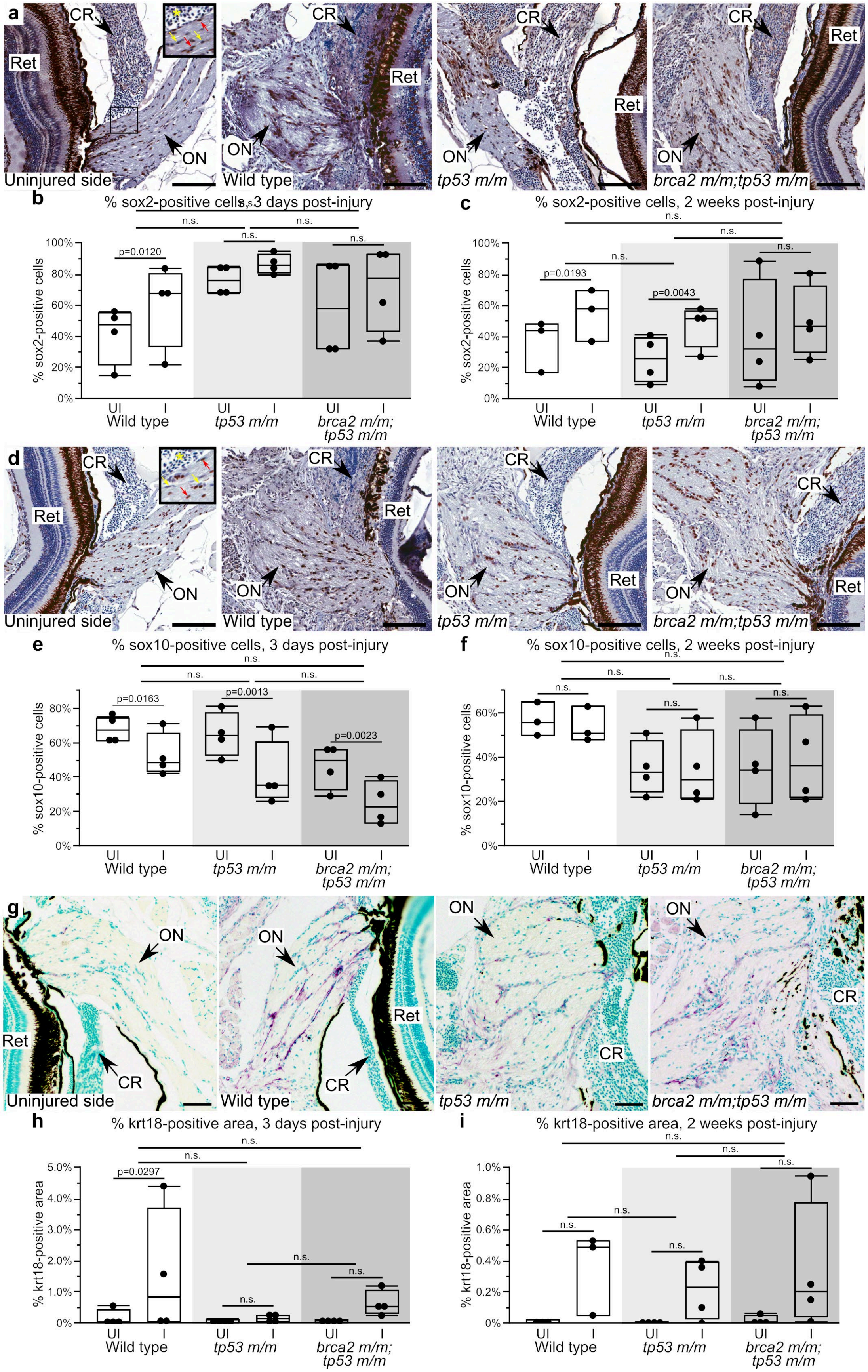

Supplementary Figure S3

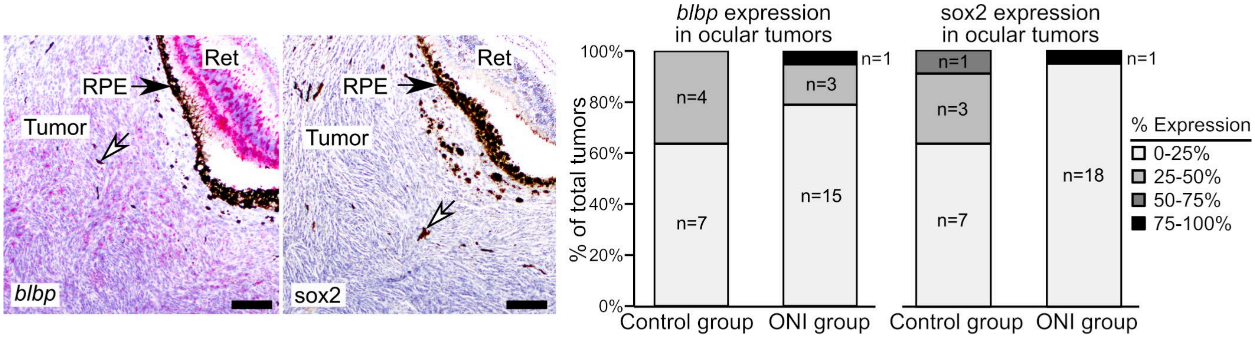

Supplementary Table S1. Statistical comparisons used to assess the relationships between genotype, optic nerve injury (ONI) status, and phenotype of ocular lesions. CR, choroid rete; mo, months. Statistically significant p-values ($p<0.05$) are in bold type.

	**Statistical test**	**p-value**
**Egg/embryo phenotype**		
Day 0, *tp53 m/m* versus *brca2 m/m;tp53 m/m*	Fisher's Exact Test	**<0.0001**
Day 1, *tp53 m/m* versus *brca2 m/m;tp53 m/m*	Fisher's Exact Test	**<0.0001**
**Site of tumor development (ocular or non-ocular)**		
ONI versus control	Fisher's Exact Test	p=0.6789
tp53 m/m ONI versus control	Fisher's Exact Test	p=0.6680
brca2 m/m;tp53 m/m ONI versus control	Fisher's Exact Test	p=1.0000
**Side of ocular tumor development (right/ONI, left/non-ONI, or bilateral)**		
ONI versus control	Fisher's Exact Test	p=0.8328
tp53 m/m ONI versus control	Fisher's Exact Test	p=0.6837
brca2 m/m;tp53 m/m ONI versus control	Fisher's Exact Test	p=0.8679
**Presence of atypical spindle cells in the CR of zebrafish without ocular tumors**[a]		
tp53 m/m versus *brca2 m/m;tp53 m/m*	Fisher's Exact Test	p=0.1941
**Presence of ocular lesion (ocular tumor, CR spindle cells) in zebrafish after ONI**[a]		
brca2 m/m;tp53 m/m versus *tp53 m/m*	Fisher's Exact Test	**p=0.0220**
**Bilateral or unilateral ocular lesion in zebrafish after ONI**[b]		
brca2 m/m;tp53 m/m versus *tp53 m/m*	Fisher's Exact Test	p=0.1207

[a] 4 zebrafish (*tp53 m/m*, n=1; *brca2 m/m;tp53 m/m*, n=3) were excluded from this comparison because the choroid rete could not be evaluated sufficiently on both sides in histologic sections.
[b] 5 zebrafish (*tp53 m/m*, n=2; *brca2 m/m;tp53 m/m*, n=3) were excluded from this comparison because the choroid rete could not be evaluated sufficiently on one side in histologic sections.

Supplementary Table S2. Statistical comparisons used to assess the relationship between optic nerve injury (ONI) status, genotype, and characteristics of the cell population in the optic nerve. I, injured; UI, uninjured. Statistically significant p-values ($p<0.05$) are in bold type.

Comparison	p-value	Difference and confidence interval
Total cellularity, three days post-injury		
Wild type (I vs. UI)	0.9554	Difference, -0.01; Lower, -0.46; Upper, 0.43
tp53 m/m (I vs. UI)	0.6805	Difference, 0.09; Lower, -0.35; Upper, 0.53
brca2 m/m;tp53 m/m (I vs. UI)	**0.0001**	Difference, 0.95; Lower, 0.56; Upper, 1.34
Wild type (I-UI) vs. *tp53 m/m* (I-UI)	0.7408	Difference, -0.10; Lower, -0.73; Upper, 0.53
Wild type (I-UI) vs. *brca2 m/m;tp53 m/m* (I-UI)	**0.0028**	Difference, -0.96; Lower, -1.55; Upper, -0.37
tp53 m/m (I-UI) vs. *brca2 m/m;tp53 m/m* (I-UI)	**0.0064**	Difference, -0.86; Lower, -1.45; Upper, -0.27
Total cellularity, two weeks post-injury		
Wild type (I vs. UI)	**0.0464**	Difference, 0.69; Lower, 0.01; Upper, 1.36
tp53 m/m (I vs. UI)	**0.0130**	Difference, 0.91; Lower, 0.24; Upper, 1.58
brca2 m/m;tp53 m/m (I vs. UI)	**0.0000**	Difference, 1.83; Lower, 1.23; Upper, 2.43
Wild type (I-UI) vs. *tp53 m/m* (I-UI)	0.6107	Difference, -0.22; Lower, -1.18; Upper, 0.73
Wild type (I-UI) vs. *brca2 m/m;tp53 m/m* (I-UI)	**0.0179**	Difference, -1.15; Lower, -2.05; Upper, -0.24
tp53 m/m (I-UI) vs. *brca2 m/m;tp53 m/m* (I-UI)	**0.0463**	Difference, -0.92; Lower, -1.82; Upper, -0.02
blbp expression, three days post-injury		
Wild type (I vs. UI)	**0.0039**	Difference, 0.05; Lower, 0.02; Upper, 0.09
tp53 m/m (I vs. UI)	**0.0179**	Difference, 0.04; Lower, 0.01; Upper, 0.07
brca2 m/m;tp53 m/m (I vs. UI)	**0.0001**	Difference, 0.09; Lower, 0.06; Upper, 0.13
Wild type (I-UI) vs. *tp53 m/m* (I-UI)	0.5134	Difference, 0.01; Lower, -0.03; Upper, 0.06
Wild type (I-UI) vs. *brca2 m/m;tp53 m/m* (I-UI)	0.0757	Difference, -0.04; Lower, -0.08; Upper, 0.01
tp53 m/m (I-UI) vs. *brca2 m/m;tp53 m/m* (I-UI)	**0.0249**	Difference, -0.05; Lower, -0.10; Upper, -0.01
blbp expression, two weeks post-injury		
Wild type (I vs. UI)	0.9207	Difference, 0.00; Lower, -0.08; Upper, 0.09
tp53 m/m (I vs. UI)	0.7072	Difference, 0.01; Lower, -0.06; Upper, 0.08
brca2 m/m;tp53 m/m (I vs. UI)	**0.0071**	Difference, 0.11; Lower, 0.04; Upper, 0.18
Wild type (I-UI) vs. *tp53 m/m* (I-UI)	0.8637	Difference, -0.01; Lower, -0.12; Upper, 0.10
Wild type (I-UI) vs. *brca2 m/m;tp53 m/m* (I-UI)	0.0526	Difference, -0.11; Lower, -0.22; Upper, 0.00
tp53 m/m (I-UI) vs. *brca2 m/m;tp53 m/m* (I-UI)	0.0533	Difference, -0.10; Lower, -0.20; Upper, 0.00
lcp1 expression, three days post-injury		
Wild type (I vs. UI)	0.7887	Difference, 0.00; Lower, 0.00; Upper, 0.00
tp53 m/m (I vs. UI)	0.1904	Difference, 0.00; Lower, 0.00; Upper, 0.00
brca2 m/m;tp53 m/m (I vs. UI)	**0.0374**	Difference, 0.00; Lower, 0.00; Upper, 0.01
Wild type (I-UI) vs. *tp53 m/m* (I-UI)	0.4409	Difference, 0.00; Lower, -0.01; Upper, 0.00
Wild type (I-UI) vs. *brca2 m/m;tp53 m/m* (I-UI)	0.1605	Difference, 0.00; Lower, -0.01; Upper, 0.00
tp53 m/m (I-UI) vs. *brca2 m/m;tp53 m/m* (I-UI)	0.4878	Difference, 0.00; Lower, 0.00; Upper, 0.00
lcp1 expression, two weeks post-injury		
Wild type (I vs. UI)	0.9935	Difference, 0.00; Lower, -0.01; Upper, 0.01
tp53 m/m (I vs. UI)	0.8692	Difference, 0.00; Lower, -0.01; Upper, 0.01
brca2 m/m;tp53 m/m (I vs. UI)	**0.0372**	Difference, 0.01; Lower, 0.00; Upper, 0.02
Wild type (I-UI) vs. *tp53 m/m* (I-UI)	0.9190	Difference, 0.00; Lower, -0.01; Upper, 0.01
Wild type (I-UI) vs. *brca2 m/m;tp53 m/m* (I-UI)	0.1422	Difference, -0.01; Lower, -0.02; Upper, 0.00
tp53 m/m (I-UI) vs. *brca2 m/m;tp53 m/m* (I-UI)	0.1386	Difference, -0.01; Lower, -0.02; Upper, 0.00

Supplementary Table S3. Statistical comparisons used to assess the relationship between optic nerve injury (ONI) status, genotype, and characteristics of the cell population in the optic nerve. I, injured; UI, uninjured. Statistically significant p-values ($p<0.05$) are in bold type.

Comparison	p-value	Difference and confidence interval
sox2 expression, three days post-injury		
Wild type (I vs. UI)	**0.0120**	Difference, 0.19; Lower, 0.05; Upper, 0.33
tp53 m/m (I vs. UI)	0.1205	Difference, 0.10; Lower, -0.03; Upper, 0.24
brca2 m/m;tp53 m/m (I vs. UI)	0.0708	Difference, 0.12; Lower, -0.01; Upper, 0.26
Wild type (I-UI) vs. *tp53 m/m* (I-UI)	0.3416	Difference, 0.09; Lower, -0.11; Upper, 0.28
Wild type (I-UI) vs. *brca2 m/m;tp53 m/m* (I-UI)	0.4621	Difference, 0.07; Lower, -0.13; Upper, 0.26
tp53 m/m (I-UI) vs. *brca2 m/m;tp53 m/m* (I-UI)	0.8187	Difference, -0.02; Lower, -0.21; Upper, 0.17
sox2 expression, two weeks post-injury		
Wild type (I vs. UI)	**0.0193**	Difference, 0.19; Lower, 0.04; Upper, 0.34
tp53 m/m (I vs. UI)	**0.0043**	Difference, 0.22; Lower, 0.09; Upper, 0.35
brca2 m/m;tp53 m/m (I vs. UI)	0.1262	Difference, 0.10; Lower, -0.03; Upper, 0.22
Wild type (I-UI) vs. *tp53 m/m* (I-UI)	0.7163	Difference, -0.03; Lower, -0.23; Upper, 0.16
Wild type (I-UI) vs. *brca2 m/m;tp53 m/m* (I-UI)	0.3079	Difference, 0.09; Lower, -0.10; Upper, 0.29
tp53 m/m (I-UI) vs. *brca2 m/m;tp53 m/m* (I-UI)	0.1521	Difference, 0.12; Lower, -0.06; Upper, 0.31
sox10 expression, three days post-injury		
Wild type (I vs. UI)	**0.0163**	Difference, -0.15; Lower, -0.27; Upper, -0.04
tp53 m/m (I vs. UI)	**0.0013**	Difference, -0.24; Lower, -0.35; Upper, -0.12
brca2 m/m;tp53 m/m (I vs. UI)	**0.0023**	Difference, -0.22; Lower, -0.33; Upper, -0.10
Wild type (I-UI) vs. *tp53 m/m* (I-UI)	0.2688	Difference, 0.09; Lower, -0.08; Upper, 0.25
Wild type (I-UI) vs. *brca2 m/m;tp53 m/m* (I-UI)	0.3986	Difference, 0.06; Lower, -0.10; Upper, 0.23
tp53 m/m (I-UI) vs. *brca2 m/m;tp53 m/m* (I-UI)	0.7767	Difference, -0.02; Lower, -0.19; Upper, 0.14
sox10 expression, two weeks post-injury		
Wild type (I vs. UI)	0.6868	Difference, -0.03; Lower, -0.19; Upper, 0.13
tp53 m/m (I vs. UI)	0.9998	Difference, 0.00; Lower, -0.14; Upper, 0.14
brca2 m/m;tp53 m/m (I vs. UI)	0.5498	Difference, 0.04; Lower, -0.10; Upper, 0.17
Wild type (I-UI) vs. *tp53 m/m* (I-UI)	0.7601	Difference, -0.03; Lower, -0.24; Upper, 0.18
Wild type (I-UI) vs. *brca2 m/m;tp53 m/m* (I-UI)	0.4892	Difference, -0.07; Lower, -0.27; Upper, 0.14
tp53 m/m (I-UI) vs. *brca2 m/m;tp53 m/m* (I-UI)	0.6705	Difference, -0.04; Lower, -0.23; Upper, 0.16
krt18 expression, three days post-injury		
Wild type (I vs. UI)	**0.0297**	Difference, 0.01; Lower, 0.00; Upper, 0.03
tp53 m/m (I vs. UI)	0.8537	Difference, 0.00; Lower, -0.01; Upper, 0.01
brca2 m/m;tp53 m/m (I vs. UI)	0.3186	Difference, 0.01; Lower, -0.01; Upper, 0.02
Wild type (I-UI) vs. *tp53 m/m* (I-UI)	0.1253	Difference, 0.01; Lower, 0.00; Upper, 0.03
Wild type (I-UI) vs. *brca2 m/m;tp53 m/m* (I-UI)	0.3092	Difference, 0.01; Lower, -0.01; Upper, 0.03
tp53 m/m (I-UI) vs. *brca2 m/m;tp53 m/m* (I-UI)	0.5554	Difference, 0.00; Lower, -0.02; Upper, 0.01
krt18 expression, two weeks post-injury		
Wild type (I vs. UI)	0.0746	Difference, 0.00; Lower, 0.00; Upper, 0.01
tp53 m/m (I vs. UI)	0.1891	Difference, 0.00; Lower, 0.00; Upper, 0.01
brca2 m/m;tp53 m/m (I vs. UI)	0.0636	Difference, 0.00; Lower, 0.00; Upper, 0.01
Wild type (I-UI) vs. *tp53 m/m* (I-UI)	0.5591	Difference, 0.00; Lower, 0.00; Upper, 0.01
Wild type (I-UI) vs. *brca2 m/m;tp53 m/m* (I-UI)	0.8916	Difference, 0.00; Lower, 0.00; Upper, 0.01
tp53 m/m (I-UI) vs. *brca2 m/m;tp53 m/m* (I-UI)	0.6262	Difference, 0.00; Lower, -0.01; Upper, 0.00

Supplementary Table S4. Experimental groups. ONI, optic nerve injury; dpi, days post-injury; wpi, weeks post-injury; IHC, immunohistochemistry; ISH, in situ hybridization; mo, months.

Evaluation of ovaries

Genotype	7.5 mo
Wild type	4
tp53 m/m	4
brca2 m/m;tp53 m/m	4

Serial sacrifice group

Genotype	3 mo	4 mo	5.1 mo[a]	5.5 mo[a]	6 mo	7 mo
Wild type	--	--	4	4	--	4
brca2 m/m	--	--	3	3	--	4
tp53 m/m	4	4	4	4	4	4
brca2 m/m;tp53 m/m	4	4	4	4	4	4

ONI group for short-term injury response

Genotype	3 dpi (ONI at 5 mo)	2 wpi (ONI at 5 mo)
Wild type	7	4
tp53 m/m	7	4
brca2 m/m;tp53 m/m	9	5

ONI group for tumorigenesis studies

Genotype	Total zebrafish[a]	Zebrafish with tumors[b]
Wild type	36	0 (0%)
tp53 m/m	54	47 (87%)
brca2 m/m;tp53 m/m	60	56 (93%)

Control group for tumorigenesis studies

Genotype	Total zebrafish[c,d]	Zebrafish with tumors[e]
tp53 m/m	55	48 (87%)
brca2 m/m;tp53 m/m	49	46 (94%)

ONI group for IHC and ISH analysis of ocular tumors

Genotype	Total tumors	Right (ONI side)	Left (non-ONI side)	Bilateral
tp53 m/m	7	3	3	1
brca2 m/m;tp53 m/m	12	5	3	4

Control group for IHC and ISH analysis of ocular tumors[f]

Genotype	Total tumors	Right (ONI side)	Left (non-ONI side)	Bilateral
tp53 m/m	5	2	3	0
brca2 m/m;tp53 m/m	6	2	4	0

[a] 8 zebrafish (*tp53 m/m*, n=3; *brca2 m/m;tp53 m/m*, n=5) were excluded from this group (see Methods)
[b] 18 zebrafish (*tp53 m/m*, n=6; *brca2 m/m;tp53 m/m*, n=12) developed two anatomically distinct tumors (e.g, an ocular tumor and a non-ocular tumor). In these cases, tumors were counted separately.
[c] The control group is a single clutch of zebrafish siblings that was previously described in an analysis of tumor ploidy, and includes all tumors identified in this population. Not all tumors from this population were included in the previous study (e.g, excluded because ploidy analysis did not pass QC analysis)[27].
[d] 11 zebrafish (*tp53 m/m*, n=6; *brca2 m/m;tp53 m/m*, n = 5) were excluded from this group (see Methods)
[e] 12 zebrafish (*tp53 m/m*, n=3; *brca2 m/m;tp53 m/m*, n=9) developed two anatomically distinct tumors (e.g, an ocular tumor and a non-ocular tumor). In these cases, tumors were counted separately.
[f] Ocular tumors were derived from a population of zebrafish with *brca2* and *tp53* mutations that were collected and processed at the same time as the ONI group in order to minimize differences in tissue orientation, fixation, processing, and specimen age.
